# Comparison between Standard Expository Cognitive Behavioral Therapy (CBT-E) and Immersive Virtual Reality CBT (CBT-VR) for Rehabilitation of Patients Affected by Occupational Stress Disorders: Study Protocol

**DOI:** 10.3390/ijerph20095735

**Published:** 2023-05-06

**Authors:** Rodolfo Buselli, Martina Corsi, Antonello Veltri, Riccardo Marino, Fabrizio Caldi, Paolo Del Guerra, Giovanni Guglielmi, Camilla Tanca, Massimo Paoli, Vincenzo Maria Calabretta, Salvio Perretta, Rudy Foddis, Marcello Antonio Carrozzino

**Affiliations:** 1 Occupational Health Department, Azienda Ospedaliero-Universitaria Pisana, Via Paradisa 2, Cisanello, 56124 Pisa, Italy; 2 Center for Work-Related Stress and Occupational Mental Disorders, Azienda Ospedaliero-Universitaria Pisana, Via Paradisa 2, Cisanello, 56124 Pisa, Italy; 3 Department of Translational Research and New Technologies in Medicine and Surgery, University of Pisa, 56126 Pisa, Italy; 4 Department of Public Health, Azienda USL Toscana Centro, 50053 Empoli, Italy; 5 Scuola Superiore Sant’Anna, Institute of Communication, Information and Perception Technologies TECIP, 56100 Pisa, Italy; 6 Department of Public Health, Sovrintendenza Sanitaria Regionale Inail Toscana, 50144 Firenze, Italy; 7 Department of Public Health, Azienda USL Toscana Centro, 59100 Prato, Italy

**Keywords:** work-related stress, occupational stress, anxiety, depression, adjustment disorder, cognitive-behavior therapy, virtual reality

## Abstract

Work-related stress presents a significant impact on work performance and physical health. It has been associated with the onset of a multitude of symptoms that can lead to occupational stress diseases, namely Adjustment Disorder and Post-Traumatic Stress Disorder. The literature has evidenced that “exposure therapy” of cognitive-behavioral training (CBT-E) seems to be the most effective technique to manage stress symptoms, including work stress diseases, and several studies have considered Virtual Reality (VR) as an adjuvant tool to exposure-based psychotherapy (CBT-VR) for the treatment of multiple psychiatric disorders. The aim of this study is to evaluate the effectiveness of CBT with exposure to stressful work scenarios in imaginative (CBT-E) and in immersive virtual reality (CBT-VR) scenarios in a group of workers affected by work-related stress disorders and compare the clinical and physiological outcomes between the two exposure techniques. A long-term goal would be to develop an evidence-based rehabilitation program as a treatment for the reintegration into work of patients affected by these psychiatric disorders.

## 1. Introduction

### 1.1. State of the Art and Rationale of the Study

According to a definition provided by the National Institute for Occupational Safety and Health, work-related stress can be defined as “harmful physical and emotional responses that occur when the requirements of the job do not match the capabilities, resources, or needs of the worker. Work-related stress can adversely affect health and even lead to injury” [1]. In Europe, 147 million workers reported that they work very tight schedules and are required to meet pressing deadlines [2,3,4]. Work-related stress affects about 22% of workers in Europe. In Italy, the number is 27%. Overall, 50% of working days lost in a year are related to work-related stress. The European Union has estimated the economic cost of work-related stress to be approximately EUR 265 billion per year [5,6].

The studies conducted so far on the factors of one’s working environment that generate stress have dealt with a complex reality that is difficult to break down into its specific features [7,8,9]. In the 1970s, Karasek’s “job strain” model stated that the greatest risk to physical and mental health from stress occurs in workers facing high psychological workload demands or pressures combined with low control or decision latitude in meeting those demands [9,10]. In 1991, Kasl produced an analytical list of the different aspects of work involved in the production of emotional distress and stress. Work stress can be caused by different factors, such as the content of the work, its organization, the environment, poor communication, etc. [11]. In October 2004, the four major European organizations in the field (the European Trade Union Confederation (ETUC), Union of Industrial and Employers’ Confederations of Europe (UNICE), European Association of Craft, Small and Medium-sized Enterprises (UEAPME), and Centre of Employers and Enterprises providing Public services (CEEP) signed an agreement on the significance of work-related stress, the policies to prevent it, and the psychiatric diseases derived from it (European Agreement on Work-Related Stress, Brussels, 2004) [12].

What has emerged to date is that work-related stress disorders are the diseases in which occupational factors interact with non-occupational factors and individual conditions [13]. Consistently, in the presence of a worker with anxiety and/or mood symptoms related to work problems, it is necessary to carry out a careful multidisciplinary evaluation of the organization of work, the personal global functioning, and personality traits aimed at recognizing the eventuality of an occupational disease due to work-related stress, such as Adjustment Disorder (AD) or Post Traumatic Stress Disorder (PTSD). The first occurs in response to one or more non-extreme stressors, while the second represents the response that may occurs in people who have experienced or witnessed a life-threatening traumatic event or a catastrophic situation [14,15,16,17].

AD is a frequent diagnosis among workers and is characterized by heterogeneous emotional or behavioral (but also somatic) symptoms, related to traumatic and stressful events, which arise in the three months following one or more work stressful events or organizational conditions. Once the stressor has terminated, the symptoms may resolve within six months (Acute Adjustment Disorder) or may persist for a longer period if the stressor has long-term consequences. Workplace exposure to serious traumatic events can also cause PTSD, a clinical condition characterized by more specific symptoms of re-experiencing, avoidance, hyperarousal, and negative alterations of cognition and mood in relation to traumatic events [18]. 

Randomized controlled trials regarding interventions aimed at reducing work-related stress indicate that cognitive behavioral therapy (CBT) is more effective than other interventions [19,20]. Throughout the literature, CBT-based interventions have been effective in improving the perceived quality of working life, improving psychological resources and responses, reducing distress, decreasing perceived stress, and decreasing symptoms of anxiety and depression [21,22]. CBT is a type of psychotherapeutic treatment that helps people to identify and change destructive or disturbing thought patterns that have a negative influence on their behavior and emotions. Under stressful conditions, some individuals tend to feel pessimistic and unable to solve problems. CBT promotes more balanced thinking to improve the ability to cope with stress [23]. These CBT interventions typically range from hours-long workshops to year-long interventions [24,25]. Van der Klink’s group (2001) [26] found an inverse relationship between the number of sessions and the effect size of CBT. This was reported in a quantitative meta-analysis evaluating 48 experimental studies. Ulter, Richardson, and Rothstein (2008) [24] have suggested that shorter programs may be more practical and cheaper to implement than longer ones. 

The literature has evidenced that “exposure therapy” of CBT (CBT-E) seems to be the most effective technique to manage stress symptoms, including work stress diseases [27]. The overall goal of exposure therapy, according to the American Psychiatric Association, is to “reduce a patient’s fear and decrease avoidance” of a patient to his “feared objects, activities, or situations” using a safe environment [28]. There are many different approaches to exposure therapy, which incorporates relaxation exercises as well as gradual exposure to the feared item or situation which tackles the person’s milder fears first before working on the more difficult tasks. In certain situations, the “flooding technique” of exposure therapy (as opposed to a gradual approach) is used to treat the most difficult or intense fear or phobia, with therapy continuing until that fear or phobia is overcome. In this treatment, patients are not allowed to remove themselves from the situation [29]. The advantage of this approach is that it is quick and usually effective. The practice of flooding, also known as “immersion,” initially produces an anxious response of maximum intensity and, as a consequence of repeated exposure to the phobic stimulus, is subsequently followed by a physiological decrease. In reality, the subject tends to deliberately and consecutively avoid the confrontation with the anxiety-provoking stimulation, thus preventing the extinction of the phobic response. Through the flooding procedure, which is one of the so-called “strong” techniques of CBT, the therapist leads the patient through the experience, on an imaginative or real level, of the situation that scares the patient the most and for as long as possible. Generally, anxiety decreases after reaching a peak or, in some cases, disappears completely [30].

It is in this context that virtual reality treatments have been introduced. Virtual reality, or VR, is the simulation of a real situation with which a subject can interact using extremely sophisticated interfaces [31,32]. The technique can simulate flooding but with the use of advanced technology. As far as the technological aspect is concerned, VR consists of tools capable of acquiring information on the subject’s actions, which are integrated and updated by the computer in real time to build a dynamic three-dimensional world. Numerous studies have highlighted that VR can be a valid tool for CBT, allowing the patient to overcome certain obstacles, especially in the case of disorders whose treatment is based on “flooding” techniques with gradual immersive exposure, allowing experiences otherwise almost impossible, if not imaginatively (CBT-E). Using VR as a therapeutic tool has many advantages (CBT-VR). Therapy mediated by VR desensitizes the subjects from their anxieties, gradually accustoming them to emotions that they can try to manage through a different approach. Furthermore, the different components of the virtual environment are entirely under the control of the therapist, so as to allow him/her to establish, from time to time, the degree of difficulty to present to the patient. In this way, the therapist plays the role of mediator between the real and virtual world [33,34,35].

Several reviews and meta-analyses have considered VR as an adjuvant tool to exposure-based psychotherapy for the treatment of multiple anxiety disorders [34,35]. Virtual environments allow the psychotherapist to actively control multiple aspects of the stimuli presented and to identify parameters related to the dysfunctional response. These environments also guarantee the confidentiality and safety of patients. Numerous meta-analyses have shown that virtual environments evoke the same reactions and emotions as the situation experienced in the real world, that the sense of presence can also be experienced in environments characterized by a graphic realism that is not particularly accurate, and that they are strongly correlated with the possibility of interacting with the components of the virtual environment [34,35]. Many studies have used VR as a simulation, interaction, and distraction tool for patients with psychiatric illnesses such as PTSD, anxiety, specific phobia, schizophrenia, autism, and dementia. In this regard, VR environments offers a possibility of exposing patients to environments that can contribute to change patients’ emotions, cognitions, and behaviors and can thus be an important tool for psychiatric rehabilitation [35]. Nevertheless, there is a literature gap regarding the application of VR in the context of work-related stress disorders. Studies on this topic have focused on the application of VR in the workplace for stress management as a tool for relaxation and not as a technique of immersing oneself in a work stressful experience to promote a process of reorganization of cognitive schemas and emotional responses from the rehabilitation perspective [36]. 

Accordingly, we believe that it is important for occupational teams to explore this kind of application in the context of work-related stress disorders. The working community lacks standardized and proven rehabilitation protocols for workers affected by work-related stress disorders. 

The aim of this study is to evaluate the effectiveness of cognitive-behavioral training (CBT) with exposure to stressful work scenarios in imaginative (CBT-E) and in immersive virtual reality (CBT-VR) scenarios in a group of workers affected by work-related stress disorders and compare the clinical and physiological outcomes between the two exposure techniques. A long-term goal would be to develop an evidence-based rehabilitation program as a treatment for the reintegration into work of patients affected by these psychiatric disorders. In the future, a short, focused, and standardized rehabilitation process aimed at improving the symptoms of anxiety and depression could, in our opinion, contribute to globally reduce working days lost.

### 1.2. Study Hypotheses

**Figure 1:**
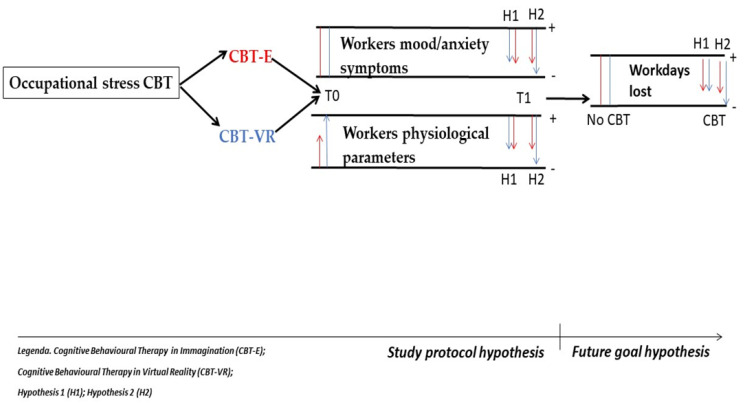
Theoretical model of the study protocol.

Taking into consideration previous literature evidence where metanalyses [26,36] have underlined the greater effect of CBT on occupational stress both on psychological and physiological outcomes than other techniques as relaxation or organizational protocols, we expect a global positive influence of both CBT practices on workers’ mental health outcomes in term of a reduction of mood and anxiety symptoms, as well as a reduction of the physiological stress response (heart rate, respiratory rate, skin conductance, and body temperature) at the end of the whole training (Hypothesis 1). Nevertheless, the single term of the impact is unpredictable because the literature studies are very heterogenous. We hypothesize a greater efficacy from the CBT-VR because the rehabilitation program will be focused on imaginative techniques with gradual desensitization, and it is assumed that VR will allows a better immersive experience. In this regard, we predict greater levels of the physiological parameters during the first session of CBT-VR, as well as greater reduction during the last session with respect to CBT-E (Hypothesis 2). Further, regarding the future application of the method, we believe that work stress disease rehabilitation with CBT-VR could have a better impact in terms of the reduction of workdays lost in line with the improvement of clinical parameters and a better modulation of stress physiological parameters (Hypothesis 2). See Figure 1.

## 2. Methods

### 2.1. Study Design, Setting, and Timeline

**Figure 2:**
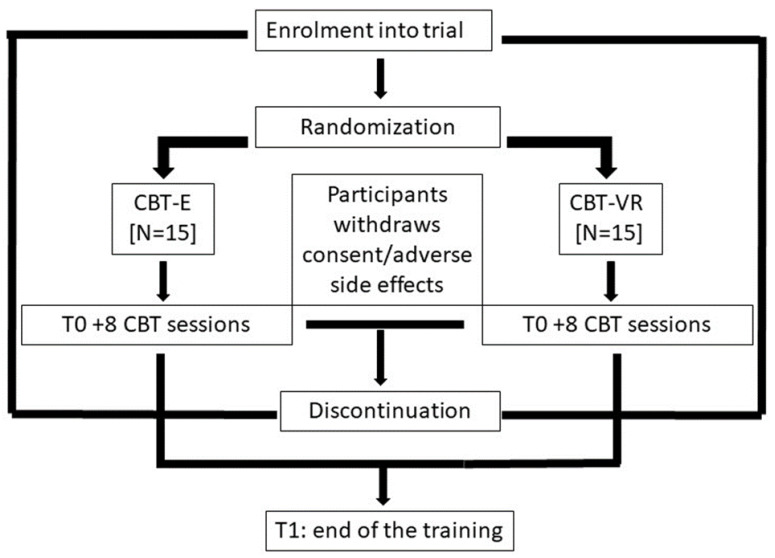
Study procedures.

The study is a controlled, randomized, non-profit parallel group superiority clinical trial fulfilling the CONSORT criteria for non-pharmacological treatment. See Figure 2. The estimated time for the entire duration of the study is 13 months as summarized in the Gantt Chart. See Table 1.

A total of 30 patients will be allocated to either CBT-E or CBT-VR. We opted for 15 patients for each group. Based on a sample of 100 healthcare workers of our University Hospital suffering from Adjustment Disorders and treated with CBT at our center (pre-treatment CGI score = 3.95 ± 0.74 and post-treatment CGI score = 2.39 ± 1.07, unpublished data), to point out a difference between the 2 treatment groups (CBT-E and CBT-VR) of at least 1 point at the CGI, the estimated sample size should be at least 26, with 13 patients in the CBT-E group and 13 patients in the CBT-VR group (by considering confidence interval = 95%, statistical power = 90%, ratio of sample size of 1). 

Participants will be assessed at baseline, during each session of CBT, and at the end of the training. Recruitment will be carried out at the outpatient care setting of INAIL, the Italian National Institute for Insurance against Accidents at Work, a public entity safeguarding workers against physical injuries and occupational diseases. The randomized clinical trial will then be conducted at the Center for Work-Related Stress and Occupational Mental Disorders of a major University Hospital in central Italy (Azienda Ospedaliero-Universitaria Pisana, AOUP). The Center includes a multidisciplinary team comprising occupational physicians, psychiatrists, and psychologists. A Trial Management Team (TMT) will be formed to conduct the day-to-day management of the trial. This will include the chief investigator, trial physicians with different roles, and the data manager. The group will meet at least once per week to discuss issues related to the progress of the trial and to ensure the trial is running well.

### 2.2. Eligibility Criteria, Tools, and Informed Consent

Recruited subjects will be workers with a diagnosis of AD or PTSD determined by means of the presence of items corresponding to DSM-5 criteria for the disorders. Inclusion and exclusion criteria for randomization in the arms of the study are summarized in Table 2. A case report form will be fulfilled for each participant reporting personal data at the beginning of the training and physiological and clinical data at baseline (T0), at every CBT session, and at the end of the training (T1). Personal background, physiological, and clinical data are summarized in Table 3.

Regarding informed consent, a first information sheet will be handed out at the outpatient clinic of INAIL during the recruitment phase. Subsequently, trial assessors of the Center for Work-Related Stress and Occupational Mental Disorders will discuss the trial, with referred patients considering the information provided in the information sheet. The assessors will then obtain written consent from patients willing to participate in the trial.

### 2.3. Measures

The whole sample will be investigated by means of the BDI-II, SAS, JCQ, PSQI, PDI, DTS, and CGI-I.

The BDI-II consists of 21 groups of statements and is one of the most widely used psychometric tests for measuring the severity of depression. This questionnaire presents statements that describes the way the patient has been feeling (quotes about sadness, pessimisms, loss of pleasure, guilty feelings [..]) during the past two weeks. Answers are scored on a scale value of 0 to 3. A total score of 0–13 is considered minimal range, 14–19 is mild, 20–28 is moderate, and 29–63 is severe, e.g., 0, I do not feel sad; 1, I feel sad much of the time; 2, I am sad all the time; 3, I am so sad or unhappy that I can’t stand it. The Italian version of the BDI-II was administered to a sample of 723 college students and to a sample of 72 depressed patients. Alpha and test-retest reliabilities were computed along with associations with age and education. The BDI-II proved a reliable and valid measure of depressive symptoms in the Italian context [19].

The SAS is a 20-item self-report assessment device built to measure anxiety levels based on scoring in 4 groups of manifestations: cognitive, autonomic, motor, and central nervous system symptoms. Answering the statements, a person should indicate how much each statement applies to him or her within a period of one or two weeks prior to taking the test. Each question (“I feel tense, nervous, restless, or agitated”) is scored on a Likert-type scale of 1–4 (based on these replies: “a little of the time,” “some of the time,” “good part of the time,” “most of the time”). Some questions are negatively worded to avoid the problem of set response. The overall assessment is based on the total score. The total raw scores range from 20 to 80. The raw score then needs to be converted to an “Anxiety Index” score using the chart on the paper version of the test. The “Anxiety Index” score can then be used to determine the clinical interpretation of one’s level of anxiety: 20–44, Normal Range; 45–59, Mild to Moderate Anxiety Levels; 60–74, Marked to Severe Anxiety Levels; 75 and above, Extreme Anxiety Levels (e.g., 0, I do not feel sad; 1, I feel sad; 2, I am sad all the time and I can’t snap out of it; 3, I am so sad and unhappy that I can’t stand it). This tool has been translated and validated in many languages, including Italian. The internal consistency reliability coefficient was 80 [36].

The JCQ is a self-administered workplace environment questionnaire designed to measure social and psychological characteristics of jobs. It consists of 49 items that explore the domains of decision latitude, psychological demands, physical demands, job insecurity, and social support. The response set is designed to assess the validity of the statement about the work environment on a Likert-type scale ranging from 1 to 4 (1 = not at all, 2 = not, 3 = yes, 3 = extremely yes). Each task statement should tell what action is performed; for whom or what you do this action; what is produced by this action; and which equipment, tools, materials, work aids, and processes you use when performing this action. (e.g., How often do you perform this task? 1, Regularly—on a daily/weekly basis; 2, Periodically—on a monthly basis; 3, Infrequently—on a yearly basis). The results are used to measure the high-demand/low-control/low-support model of job strain development. Authorized translations of the full JCQ instrument from English, which have been specifically approved and which are available from the JCQ Center, are French-Canadian, French-Belgium, Flemish-Belgium, Spanish, Swedish, Dutch, Italian, and Japanese [9].

The PSQI is a 19 items questionnaire that measures several different aspects of sleep, offering seven component scores and one composite score. The component scores consist of subjective sleep quality, sleep latency (i.e., how long it takes to fall asleep), sleep duration, habitual sleep efficiency (i.e., the percentage of time in bed that one is asleep), sleep disturbances, the use of sleeping medication, and daytime dysfunction. Each item is weighted on a 0–3 interval scale. In scoring the PSQI, seven component scores are derived, each scored from 0 (no difficulty) to 3 (severe difficulty). The component scores are summed to produce a global score (range: 0–21). Higher scores indicate worse sleep quality (e.g., 0, Very good; 1, Fairly good; 2, Fairly bad; 3 Very bad). The Italian version of the questionnaire provides a good and reliable differentiation between normal and pathological groups, with higher scores reported by people characterized by impaired objectively evaluated sleep quality [37,38].

The PDI is a self-report instrument developed to retrospectively measure the distress experienced by the subject at the time of the potentially traumatizing event or immediately after. It is composed of 13 items, each scored on a 5-point Likert-type scale ranging from 0 to 4 (0 = not at all, 1 = slightly, 2 = somewhat, 3 = very, and 4 = extremely true), with the total score ranging from 0 to 52, and higher scores indicating increased distress. Statements explore cognitive response to the trauma (e.g., I thought I might die), emotional distress (e.g., I was horrified by what happened) or physical symptoms (e.g., I had physical reactions like sweating, shaking, and pounding heart). The PDI demonstrates good test–retest reliability, convergent and divergent validity, and good internal consistency. The internal consistency of the PDI was satisfactory with a Cronbach’s alpha of 0.87. The reliability, validity, internal consistency, and the temporal stability of the Italian version of the PDI resulted to be good, falling within the range of those reported in the original validation study [39].

The DTS is a self-report instrument developed to assess DSM PTSD symptoms. It is a 5-point Likert-type scale, self-report instrument that assesses the DSM symptoms of PTSD. It is composed of 17 items, each scored on a 5-point Likert-type scale evaluating frequency (0 = “not at all” to 4 = “every day”) and severity (0 = “not at all distressing” to 4 = “extremely distressing”) of the most disturbing trauma (e.g., of statements: Have you had painful images, memories, or thoughts of the event?). Respondents rate how much trouble they have had with each symptom. The scale demonstrated good test–retest reliability (r = 0·86), internal consistency (r = 0·99). The Italian version of the tool by Pieraccini et al. (1999) has been approved by the original authors [13,40].

The clinical global impression-improvement scale (CGI-I) is a 7-point scale that requires the clinician to assess how much the patient’s illness has improved or worsened relative to a baseline state at the beginning of the intervention. (e.g., clinicians ask: “Compared to the patient’s condition at baseline, this patient’s [average] condition has...?” and rate as: Very much improved; Much improved; Minimally improved; No change; Minimally worse; Much worse; Very much worse). The Italian version of the tool has been approved by the National Institute of Mental Health [41].

### 2.4. Experimental Devices Utilised

The whole sample will be monitored using Biosignalplus series devices (PLUX Wireless Biosignals S.A., Lisbon—Portugal). Four sensors for detecting heart rate, respiratory rate, skin conductance, and body temperature, designed to collect data continuously and éin real time, will be used. These devices are connected to a 4-channel hub (Biosignalsplux 4-channel hub). The hub uses a Bluetooth Class II connection to communicate with the computer and transmit captured data. The data will be processed by the dedicated OpenSignals software.

Participants in the CBT-VR arm will also wear an Oculus Quest wireless virtual reality (VR) headset. The viewer is managed by a specific application that has been developed with “multiplayer” architecture using the Unreal Engine 4 development tool and that includes an immersive application that is used by the patient through the VR viewer and a desktop application that is used by the therapist, which acts as a server and determines everything the user will see. See Table 4.

Once the application has been started on the desktop, the operator, using a third-party program (Sidequest), controls the start of the application on the user’s display, which will directly receive the data selected by the operator. Furthermore, through Sidequest, the operator will be able to monitor everything the user sees and has the ability to reset the program.

Three different standard work environments have been created: an office, a supermarket, and a mechanic’s shop.

In the first scenario, the character that interacts with the patient is sitting behind a desk with a computer. From a big window in the background, the patient can see other characters walking inside the hall next to the office. The main character wears a formal suit with a tie (or a business suit for the female character). The user’s point of view is collocated in front of the desk in a seated position.

In the second scenario, the environment represents a medium-sized supermarket with some cashiers and shoppers walking between shelves. In the background, the typical sounds of the supermarket can be heard. The character that interacts with the patient is standing in front of the checkout wearing a supermarket uniform over moderately formal clothes (e.g., shirt/tie). The user’s point of view is placed behind the checkout, as if he/she is the cashier during his/her work shift. The third environment is a standard mechanical workshop where a character appears in dirty overalls; in this scenario, the characters (mobber and patient) are simply standing in space facing each other.

The 3D characters can be male and/or female and are selected from a group of 20 model derived from real pictures of the mobbers with the possibility of adding particular features (e.g., beard, haircut). Three body animations have been created for all characters ranging from a rest position (not completely static) to a typical animated argument, both for standing and seated positions. As for the face, there is the possibility to program particular expressions to add emotion and realism to the characters using the universally recognized faces of emotions: happiness, sadness, anger, surprise, and fear, with the possibility of a six expression of “flirt”.

In this way, different working life scenarios can be created with increasing anxiety-provoking intensity.

### 2.5. Procedures, Outcomes, and Adverse Events

Participants will be randomized with a 1:1 ratio for CBT-E or CBT-VR without any specific criteria in order to prevent any randomization bias. Researchers involved in the outcome measures will be blinded to the allocation. Both the CBT training are assumed to be appropriate and beneficial for participants based on evidence from the literature. The training for each individual subject will begin within 7 days from T0, and each patient will perform 8 sessions of CBT-E/CBT-VR distributed 1 every week. If the subject is unable to perform the session on the scheduled day, a new date will be agreed within one week, failing which the patient would be excluded from the study. The intervention will also be discontinued if the participant withdraws consent or if a change in circumstance results in the participant meeting the trial exclusion criteria and becomes unsafe to engage in the intervention, or if any side effects would arise. These participants will remain in the analysis for data collected up until the date of withdrawal. The intervention is individualized, and progression/modification will be determined through discussions with the subject and the other members of the trial team. Every discontinuation will be replaced in order to reach the trial sample size.

The training protocol of CBT-E/CBT-VR is summarized in Table 5.

All outcomes will be performed before, during, and after the intervention phase, which is 10 weeks in total. The first week involves enrollment, randomization, and baseline data; the second to ninth week involves the training program; and the tenth week involves the patient final report. The outcomes measured will be physiological and clinical parameters, as well as their variations/comparison during the two training programs.

In particular, the physiological signs of stress reporting variations of temperature, skin conductance, heart rate, and respiratory rate will be recorded.

Acute psychic events (such as anxious states up to panic attacks or other emotional states of intensity that cannot be tolerated by the subject) or neurovegetative manifestations given using the 3D viewer, such as nausea, dizziness, etc., could occur during the sessions. Finally, there could be minimal traumatisms with objects in the room where the virtual simulation activity will be carried out. Such events could lead to the interruption of exposure, and, in this case, they would be managed clinically by the therapist. Whether to resume the exposure technique will be decided clinically on a case-by-case basis. Any adverse events will be collected in an ad hoc form and notified as required by current legislation.

### 2.6. Data Collection, Management, Statistical Method, and Ethical Considerations

The collection of the data will be performed in paper and later entered electronically. All data will be confidential. Enrolled patients will be registered in anonymous form using an identification code (progressive number), which will be used for data management solely for research purposes. The recorded data will be anonymized to avoid any link with patients. The data will be archived in an Excel program and kept at the trial sites under the responsibility of the principal investigator. Access to the data will be allowed only to personnel directly involved in the research protocol and protected by a password. Patients will not be identified by name in future publications. Participants will be informed about all main aspects of the research and of their right to refuse to take part or to withdraw consent to participation at any moment. An insurance policy to protect the subjects participating in the trial will be stipulated with the insurance company selected by the University of Pisa.

The data will be recorded in a specifically designed database and elaborated by means of the SPSS software (version 26). The Kolmogorov–Smirnov test will be used to examine variables for normal (Gaussian) distribution. Therefore, the comparisons between patients’ groups for variables of non-Gaussian distribution will be performed by means of non-parametric statistical tests; in particular, the Mann–Whitney test for independent samples will be used. The Student’s t-test for independent samples will be used to compare groups for variables of Gaussian distribution. According to non-Gaussian or Gaussian distribution of paired data, the Wilcoxon test and the Friedman test or the Student’s t-test for dependent samples and the repeated measures ANOVA will be used, respectively. The chi-square test will be used to compare the frequencies of categorical variables. Correlations between continuous variables will be examined by means of Spearman’s coefficient in case of non-Gaussian distribution or by means of Pearson’s coefficient in case of Gaussian distribution. A regression analysis will be used to explore the relationship between more variables. A *p* value less than 0.05 will be considered significant.

The study will be conducted in accordance with the 1964 Helsinki Declaration. Ethic codes: Helsinki, Art.3; Helsinki, Article 1; Nuremberg, Art.2; Nuremberg, Art.2; Helsinki, Articles 4, 5, 7; Helsinki, Art.6; Helsinki, Articles 9, 10, 11. The study will be also conducted in accordance to the Directive 2001/20/EC for Clinical Trials. The study protocol has been approved by the Ethics Committee of Area Vasta Nord-Ovest Toscana (Italy).

## 3. Discussion

Globally, in the literature, the application of VR has been considered in many fields, but researchers in Italy have not yet explored the possibility of using it for the widespread exposure of CBT therapy in the work context with the aim of being able to define standardizable and short rehabilitation paths for the improvement of the mental health of workers.

The outcomes of this trial will determine whether CBT rehabilitation can improve the mental health of workers suffering from occupational stress diseases. The secondary outcomes will determine the superiority of VR rehabilitation program with respect to normal exposure therapy.

In summary, the novelty of this study protocol is that, for the first time, a trial explored the efficacy of CBT rehabilitation for Italian workers affected by occupational stress diseases and attempted to determine whether CBT through virtual reality is an advantage to a classical exposure therapy. The work-related stress diseases rehabilitation program described in this protocol has been designed to be amenable to be reproduced and offered to workers. The future application is to offer workers a treatment option with evidence of effectiveness and allow a more rapid reintegration into work.

## Figures and Tables

**Table 1:** Gantt Chart.

Gantt Chart	Study Protocol Timeline							
	1–3 m	4–5 m	6–8 m	9–10 m T0 1st w	9–10 m 2–9 w CT	9–10 m T1 10th w	11–12 m	13 m
Protocol finalization								
Ethical and other reg. clearance								
Recruitment of sample for the CT								
CT intervention phase								
data collection								
Data analysis								
Dissemination								

**Table 2:** Inclusion and exclusion criteria.

*Inclusion criteria*
Diagnosis of AD or PTSD according to the DSM-5 diagnostic criteria
Age ≥18 years
Being a worker
Good understanding of verbal and written Italian
Not having other Axis I diagnoses according to DSM-5 diagnostic criteria
Being drug-free for at least 2 weeks or on stable therapy for at least 12 weeks
Having signed the informed consent to participate in the study
*Exclusion criteria*
Severe cognitive impairment as to compromise the ability to understand the aims and characteristics of the study
Suicidal ideation, neurological, vestibular, or ocular disorders and other clinical entities in the acute phase
Having undergone any CBT training in the 6 months prior to the recruitment

**Table 3:** Data collected from participants.

Demographic variables (age, gender, education level, civil status, children, family history of mental disorders, personal physical illnesses)
Work variables (field of work, public/private company, big/small company, type of contract, seniority level)
Physiological parameters (hearth rate, respiratory rate, temperature, and skin conductance)
Beck Depression Inventory-II (BDI-II), a 21-item self-report inventory measuring the severity of depression with two subscale (cognitive/affective and somatic) [19]
Self-rating Anxiety Scale (SAS), self-administered, 20-item tool for anxiety symptoms [37]
Job Content Questionnaire (JCQ), a self-administered workplace environment questionnaire to measure social and psychological characteristics of jobs with three main subscales (decision latitude, psychological job demands, and social support [9]
Pittsburgh Sleep Quality Index (PSQI), a self-rated questionnaire that assesses sleep quality and disturbances over a 1-month time interval [38]
Peritraumatic Distress Inventory (PDI), a 13-item self-report questionnaire that measures the level of distress experienced by an individual during and shortly after a traumatic event [39]
Davidson Trauma Scale (DTS), self-rating for measuring the frequency and severity of Post-traumatic Stress Disorder (PTSD) symptoms in three clusters: intrusion/avoidance/hyperarousal [13]
Clinical Global Impressions Improvments (CGI-I), to quantify patient progress/treatment response over time [40]

**Table 4:** Desktop application elements.

Setting selected in the initial menu
Gender of the character selected in the initial menu
Facial expressions selected in real time by keyboard
Body movements selected in real time by keyboard
Sentences in real time spoken by the operator and listened by the user with a voice that changes tone depending on the gender and personality of the character

**Table 5:** CBT rehabilitation protocol for occupational stress diseases.

*Session 1*	Global evaluation of the patient. Administration of psychometric instruments. Assessment and engagement, including the development of a problem list and establishment of shared goals. Introduction to the principle of exposure therapy: definition and rationale.
*Session 2*	Classification of work stimuli through a Subjective Units of Distress Scale (SUDS: 0 = no anxiety at all; completely calm; 3 = some anxiety, but manageable: 5 = getting tough; wouldn’t want to have it all the time; 7–8 = severe anxiety that interferes with daily life; 10 = worst anxiety you’ve ever felt). Analysis of some sensations high distressed stimuli/situations avoided. Discussion of catastrophic interpretations. Disadvantages resulting from the avoidance and/or application of other dysfunctional behavioral mechanisms.
*Session 3*	Exposure to a neutral scene aimed at setting the exposition in the VR environment or in the imagination. Inter-session tasks: ABC techniques for work-related emotional stimuli. Practice of relaxing exercises.
*Session 4–8*	Exercises of exposure to the maximum phobic work stimuli, at the maximum level of intensity for an extended amount of time (flooding technique) using the VR environment or in imagination alternating with relaxation exercises. Analysis of the emotions felt during the approach to the phobic stimulus with re-elaboration of it and related thoughts. Identification of dysfunctional behaviors linked to it and identification of more functional behaviors.

## Data Availability

Datasets will be available upon request to authors.

## References

[1] Tamers S.L., Streit J., Pana-Cryan R., Ray T., Syron L., Flynn M.A., Castillo D., Roth G., Geraci C., Guerin R. (2020). Envisioning the future of work to safeguard the safety, health, and well-being of the workforce: A perspective from the CDC’s National Institute for Occupational Safety and Health. Am. J. Ind. Med..

[2] Cox T., Leka S., Ivanov I., Kortum E. (2004). Work, employment and mental health in Europe. Work Stress.

[3] Moreno Fortes A., Tian L., Huebner E.S. (2020). Occupational Stress and Employees Complete Mental Health: A Cross-Cultural Empirical Study. Int. J. Environ. Res. Public Health.

[4] International Labor Organization (ILO) (2016). Workplace Stress: A Collective Challenge.

[5] European Agency for Safety and Health at Work (2014). Calculating the Cost of Work-Related Stress and Psychosocial Risks.

[6] Russo S., Ronchetti M., Di Tecco C., Valenti A., Jain A., Mennini F.S., Leka S., Iavicoli S. (2021). Developing a cost-estimation model for work-related stress: An absence-based estimation using data from two Italian case studies. Scand. J. Work. Environ. Health.

[7] EU-Osha (2000). Research on Work-Related Stress.

[8] NIOSH (1999). Stress at Work.

[9] Karasek R., Brisson C., Kawakami N., Houtman I., Bongers P., Amick B. (1998). The Job Content Questionnaire (JCQ): An instrument for internationally comparative assessments of psychosocial job characteristics. J. Occup. Health Psychol..

[10] Karasek R.A. (1979). Job demands, job decision latitude, and mental strain: Implication for job redesign. Adm. Sci. Q..

[11] Kasl S.V., Schroeder H.E. (1991). Assessing health risk in the work setting. New Directions in Health Psychology Assessment.

[12] Buselli R., Carmassi C., Corsi M., Baldanzi S., Battistini G., Chiumiento M., Massimetti G., Dell’osso L., Cristaudo A. (2021). Post-traumatic stress symptoms in an Italian cohort of subjects complaining occupational stress. CNS Spectr..

[13] Carmassi C., Bui E., Bertelloni C.A., Dell’Oste V., Pedrinelli V., Corsi M., Baldanzi S., Cristaudo A., Dell’Osso L., Buselli R. (2021). Validation of the Italian version of the peritraumatic distress inventory: Validity, reliability and factor analysis in a sample of healthcare workers. Eur. J. Psychotraumatol..

[14] Carmassi C., Pedrinelli V., Dell’Oste V., Bertelloni C.A., Cordone A., Bouanani S., Corsi M., Baldanzi S., Malacarne P., Dell’Osso L. (2021). Work and social functioning in frontline healthcare workers during the covid-19 pandemic in Italy: Role of acute post-traumatic stress, depressive and anxiety symptoms. Riv. Psichiatr..

[15] Buselli R., Veltri A., Baldanzi S., Bozzi S., Marino R., Chiumiento M., Dell’Osso L., Cristaudo A. (2016). Work-related stress disorders: Variability in clinical expression and pitfalls in psychiatric diagnosis. Med. Lav..

[16] Buselli R., Corsi M., Baldanzi S., Chiumiento M., Del Lupo E., Dell’Oste V., Bertelloni C.A., Massimetti G., Dell’Osso L., Cristaudo A. (2020). Professional Quality of Life and Mental Health Outcomes among Health Care Workers Exposed to Sars-Cov-2 (Covid-19). Int. J. Environ. Res. Public Health.

[17] American Psychiatric Association (2013). Diagnostic and Statistical Manual of Mental Disorders.

[18] Beck A.T., Steer R.A., Brown G.K. (1996). Beck Depression Inventory: Second Edition Manual.

[19] Beck A.T., Haigh E.A. (2014). Advances in cognitive theory and therapy: The generic cognitive model. Annu. Rev. Clin. Psychol..

[20] Grime P.R. (2004). Computerized cognitive behavioural therapy at work: A randomized controlled trial in employees with recent stress-related absenteeism. Occup. Med..

[21] Joyce S., Modini M., Christensen H., Mykletun A., Bryant R., Mitchell P.B., Harvey S.B. (2016). Workplace interventions for common mental disorders: A systematic meta-review. Psychol. Med..

[22] Nakao M., Shirotsuki K., Sugaya N. (2021). Cognitive-behavioral therapy for management of mental health and stress-related disorders: Recent advances in techniques and technologies. BioPsychoSoc. Med..

[23] Richardson K.M., Rothstein H.R. (2008). Effects of occupational stress management intervention programs: A meta-analysis. J. Occup. Health Psychol..

[24] Naidu V., Giblin E., Burke K.M., Madan I. (2016). Delivery of cognitive behavioural therapy to workers: A systematic review. Occup. Med..

[25] Van der Klink J.J., Blonk R.W., Schene A.H., van Dijk F.J. (2001). The benefits of interventions for work-related stress. Am. J. Public Health.

[26] Kaczkurkin A.N., Foa E.B. (2015). Cognitive-behavioral therapy for anxiety disorders: An update on the empirical evidence. Dialogues Clin. Neurosci..

[27] Huang T., Li H., Tan S., Xie S., Cheng Q., Xiang Y., Zhou X. (2022). The efficacy and acceptability of exposure therapy for the treatment of post-traumatic stress disorder in children and adolescents: A systematic review and meta-analysis. BMC Psychiatry.

[28] Foa E.B., McLean C.P. (2016). The Efficacy of Exposure Therapy for Anxiety-Related Disorders and Its Underlying Mechanisms: The Case of OCD and PTSD. Annu. Rev. Clin. Psychol..

[29] Rauch S.A., Eftekhari A., Ruzek J.I. (2012). Review of exposure therapy: A gold standard for PTSD treatment. J. Rehabil. Res. Dev..

[30] Parsons T.D., Rizzo A.A. (2008). Affective outcomes of virtual reality exposure therapy for anxiety and specific phobias: A meta-analysis. J. Behav. Ther. Exp. Psychiatry.

[31] Powers M.B., Emmelkamp P.M. (2008). Virtual reality exposure therapy for anxiety disorders: A meta-analysis. J. Anxiety Disord..

[32] Riva G. (2005). Virtual reality in psychotherapy: Review. Cyberpsychol. Behav..

[33] Rizzo A., Lange B., Suma E.A., Bolas M. (2011). Virtual reality and interactive digital game technology: New tools to address obesity and diabetes. J. Diabetes Sci. Technol..

[34] Wiederhold B.K., Wiederhold M.D. (2006). Virtual Reality as a Tool in Early Interventions.

[35] Park M.J., Kim D.J., Lee U., Na E.J., Jeon H.J. (2019). A Literature Overview of Virtual Reality (VR) in Treatment of Psychiatric Disorders: Recent Advances and Limitations. Front. Psychiatry.

[36] Zung W.W. (1971). A rating instrument for anxiety disorders. Psychosomatics.

[37] Buysse D.J., Reynolds C.F., Monk T.H., Berman S.R., Kupfer D.J. (1989). The Pittsburgh Sleep Quality Index: A new instrument for psychiatric practice and research. Psychiatry Res..

[38] Curcio G., Tempesta D., Scarlata S., Marzano C., Moroni F., Rossini P.M., Ferrara M., De Gennaro L. (2013). Validity of the Italian version of the Pittsburgh Sleep Quality Index (PSQI). Neurol. Sci..

[39] Brunet A., Weiss D.S., Metzler T.J., Best S.R., Neylan T.C., Rogers C., Fagan J., Marmar C.R. (2001). The Peritraumatic Distress Inventory: A proposed measure of PTSD criterion A2. Am. J. Psychiatry.

[40] Davidson J.R., Book S.W., Colket J.T., Tupler L.A., Roth S., David D., Hertzberg M., Mellman T., Beckham J.C., Smith R.D. (1997). Assessment of a new self-rating scale for posttraumatic stress disorder. Psychol. Med..

[41] Guy W. (1976). ECDEU Assessment Manual for Psychopharmacology.

